# Unraveling the Predictive Potential of Rapid Scoring in Pleural Infection: A Critical Review

**DOI:** 10.7759/cureus.44515

**Published:** 2023-09-01

**Authors:** Srinivasulareddy Annareddy, Babaji Ghewade, Ulhas Jadhav, Pankaj Wagh

**Affiliations:** 1 Respiratory Medicine, Jawaharlal Nehru Medical College, Datta Meghe Institute of Higher Education and Research, Wardha, IND

**Keywords:** apache ii, a-drop, curb-65, morbidity, pleural empyema, pleural infection

## Abstract

Pleural infection, or pleural empyema, is a severe medical condition associated with high morbidity and mortality rates. Timely and accurate prognostication is crucial for optimizing patient outcomes and resource allocation. Rapid scoring systems have emerged as promising tools in pleural infection prognostication, integrating various clinical and laboratory parameters to assess disease severity and quantitatively predict short-term and long-term outcomes. This review article critically evaluates existing rapid scoring systems, including CURB-65 (confusion, uremia, respiratory rate, blood pressure, age ≥ 65 years), A-DROP (age (male >70 years, female >75 years), dehydration, respiratory failure, orientation disturbance, and low blood pressure), and APACHE II (acute physiology and chronic health evaluation II), assessing their predictive accuracy and limitations. Our analysis highlights the potential clinical implications of rapid scoring, including risk stratification, treatment tailoring, and follow-up planning. We discuss practical considerations and challenges in implementing rapid scoring such as data accessibility and potential sources of bias. Furthermore, we emphasize the importance of validation, transparency, and multidisciplinary collaboration to refine and enhance the clinical applicability of these scoring systems. The prospects for rapid scoring in pleural infection management are promising, with ongoing research and data science advances offering improvement opportunities. Ultimately, the successful integration of rapid scoring into clinical practice can potentially improve patient care and outcomes in pleural infection management.

## Introduction and background

Pleural infection, commonly known as pleural empyema, is a serious medical condition characterized by the accumulation of infected fluid in the pleural space, the thin, fluid-filled cavity surrounding the lungs. It can arise as a complication of various respiratory infections, such as pneumonia, and may result from both bacterial and, less frequently, fungal pathogens. Pleural infection poses a significant health burden worldwide, with high morbidity and mortality rates, especially in vulnerable populations such as the elderly, immunocompromised individuals, and those with pre-existing lung diseases [1-4]. Managing pleural infection is complex and requires a multidisciplinary approach, including appropriate antimicrobial therapy and drainage of the infected fluid. A crucial aspect of effective management is the early identification of patients at higher risk of adverse outcomes, as this allows timely intervention and optimization of treatment strategies [5-7].

Prognostication is pivotal in determining the most appropriate therapeutic approach and predicting patient outcomes in pleural infection. Identifying patients with severe diseases and poor prognoses can aid clinicians in tailoring treatment plans and allocating resources more efficiently. Traditional prognostic indicators such as clinical signs, laboratory parameters, and radiological findings are valuable but may lack the desired accuracy to predict disease progression reliably [8-10]. The quest for accurate prognostic tools in pleural infection has led to exploring various scoring systems, aiming to integrate multiple clinical and laboratory variables to offer a more comprehensive assessment of disease severity and prognosis. These scoring systems are designed to aid physicians in risk stratification, facilitating clinical decision-making and potentially improving patient outcomes [11-13].

Rapid scoring systems have emerged as promising tools in the prognostication of pleural infection. These scoring systems leverage clinical, radiological, and laboratory parameters to generate a quantifiable score, enabling clinicians to quickly evaluate the severity of the disease and predict the likelihood of specific outcomes, such as mortality, treatment response, and length of hospital stay [14,15]. By synthesizing multiple variables into a single numerical score, rapid scoring systems aim to provide a more standardized and objective approach to prognostication. This can enhance clinical efficiency, facilitating timely interventions and potentially improving patient outcomes [16].

This review aims to comprehensively evaluate the predictive potential of rapid scoring systems in pleural infection. By critically analyzing the existing literature and summarizing the findings from various studies, we aim to provide an in-depth understanding of the utility and limitations of these scoring systems in clinical practice. We will compare their performance in predicting short-term and long-term outcomes. Additionally, we will explore the factors that may influence the accuracy of these scoring systems, such as patient demographics, microbiological and radiological findings, and the presence of comorbidities.

## Review

Overview of existing rapid scoring systems for pleural infections

In recent years, significant advancements have been made in developing and validating rapid scoring systems tailored for pleural infection prognostication. These scoring systems represent a crucial step toward enhancing clinical decision-making and optimizing patient care in the management of this complex condition. By providing a swift and reliable method to assess disease severity and predict outcomes, these rapid scoring systems offer a valuable tool for healthcare providers [17]. Three prominent rapid scoring systems commonly employed in pleural infection prognostication have emerged through our research: (i) CURB-65 (confusion, uremia, respiratory rate, blood pressure, age ≥ 65 years), (ii) A-DROP (age (male >70 years, female >75 years), dehydration, respiratory failure, orientation disturbance, and low blood pressure), and (iii) APACHE II (acute physiology and chronic health evaluation II).

The CURB-65 Score

Originally devised for predicting outcomes in community-acquired pneumonia, the CURB-65 score has been adapted and validated for use in pleural infection scenarios. It is based on five readily available clinical variables: confusion, urea level, respiratory rate, blood pressure, and age ≥65 years. By incorporating these parameters, the scoring system offers a simple yet effective approach to risk stratification and prognostication in pleural infection cases [18].

The A-DROP Score

Originally designed for predicting severe pneumonia, the A-DROP score has found relevance in the context of pleural infection prognostication. This scoring system comprises five essential parameters: age, dehydration, respiratory failure, orientation disturbance, and low blood pressure. By encompassing these key variables, the A-DROP score assists in identifying patients at higher risk of adverse outcomes, and guiding appropriate interventions [19].

The APACHE II Score

Widely employed in intensive care units, the APACHE II score is a comprehensive scoring system that incorporates various physiological parameters to assess disease severity and predict outcomes in critically ill patients, including those with pleural infection. While the APACHE II score may involve more extensive laboratory measurements and clinical evaluations, it offers a more detailed and nuanced assessment of patient status, aiding in precise prognostication [20].

These rapid scoring systems serve as valuable tools for healthcare providers, enabling them to assess disease severity and anticipate patient outcomes rapidly. These scoring systems offer a standardized and objective approach to pleural infection prognostication by integrating key clinical and laboratory parameters. By promptly identifying patients at higher risk of adverse outcomes, clinicians can optimize treatment plans, allocate resources effectively, and potentially improve patient outcomes [21].

Despite their effectiveness, it is essential to recognize that these scoring systems are adjunctive tools and should not replace clinical judgment. The context of each patient's presentation, comorbidities, and response to therapy remains crucial in the overall management of pleural infection. Continued research, validation, and refinement of these rapid scoring systems are vital for maximizing their clinical utility and further improving patient care in pleural infection management. With ongoing efforts and multidisciplinary collaboration, rapid scoring systems promise to positively impact pleural infection management in the future [22].

Comparison of different scoring systems

Scoring Criteria and Components

Scoring criteria and components form the foundation of rapid scoring systems in pleural infection prognostication, as they determine the variables used to generate a numerical score reflecting disease severity. These variables' selection varies among scoring systems based on their intended scope and underlying concepts [14].

Some rapid scoring systems like CURB-65 and A-DROP utilize more straightforward and easily accessible clinical parameters to calculate the score. These criteria typically include age, respiratory rate, blood pressure, and mental status. These variables can be quickly assessed at the bedside and are commonly available in most healthcare settings. By focusing on straightforward clinical indicators, these scoring systems offer a convenient and time-efficient approach to prognostication, particularly in resource-limited environments [23].

On the other hand, the APACHE II score takes a more comprehensive approach by integrating a wide range of physiological and laboratory measurements. These variables encompass arterial pH, oxygenation, serum creatinine levels, and hematocrit. The APACHE II score was originally designed for assessing the severity of critically ill patients in intensive care units and has been adapted for use in various clinical settings, including pleural infection. Including a broader array of physiological parameters enhances the APACHE II score's discriminative ability, allowing for a more detailed evaluation of disease status and patient outcomes [24].

The choice between simpler clinical variables and more complex physiological measurements depends on the scoring system's intended use and clinical context. While rapid scoring systems like CURB-65 and A-DROP offer practicality and ease of use, the APACHE II score provides a more nuanced assessment, especially for critically ill patients. When selecting the most appropriate rapid scoring approach for pleural infection prognostication, healthcare providers must consider the scoring system's applicability to their specific patient population and available resources. These scoring criteria and components are essential for risk stratification and clinical decision-making, contributing to improved patient care and outcomes [25].

Pros and Cons of Each Scoring System

The CURB-65 and A-DROP scores offer practical benefits due to their straightforwardness and ease of calculation. These scores utilize easily obtainable clinical parameters, including age, respiratory rate, blood pressure, and mental status, which can be swiftly evaluated at the patient's bedside. Their limited reliance on extensive laboratory tests makes them particularly suitable for settings with limited resources, where access to advanced medical facilities might be constrained. Furthermore, their uncomplicated nature allows for rapid risk assessment, enabling timely interventions in patients with pleural infection. However, the simplicity of the CURB-65 and A-DROP scores might result in some degree of oversimplification in disease evaluation. The sensitivity, specificity, positive predictive value, and negative predictive value of CURB-65 were 71%, 69%, 35%, and 91%, respectively. The best-performing version of the systemic inflammatory response syndrome (SIRS) criteria showed rates of 62%, 73%, 35%, and 89%, respectively, while the standardized early warning scoring system (SEWS) exhibited rates of 52%, 67%, 27%, and 86%, respectively. By concentrating on a restricted set of criteria, these scoring systems may not fully encompass the intricacies and variations present in cases of pleural infection. This limitation could potentially impact their accuracy in predicting outcomes [26].

On the contrary, the APACHE II score provides a more advanced and comprehensive evaluation of a patient's condition, which holds particular significance for those critically ill with pleural infections. The APACHE-II score demonstrated a sensitivity of 89.9% and a specificity of 97.6%. By amalgamating various physiological measurements and laboratory results, the APACHE II score delivers a thorough assessment of the severity of the disease and the dysfunction of organs. This renders it especially pertinent within intensive care units (ICUs) confines. Its capacity to consider multiple variables enables a more subtle and precise classification of risk, thereby assisting healthcare practitioners in customizing treatment strategies for critically ill patients. Nevertheless, the reliance on the APACHE II score on multiple intricate laboratory measurements and complex computations could impede its immediate application within standard clinical practice, particularly in non-ICU environments. In scenarios with limited time and resources, the extensive data collection demanded by the APACHE II score might not always be feasible or pragmatic. Consequently, this could diminish its practicality and clinical influence [27].

Current Challenges in Prognostication Using Rapid Scoring

While rapid scoring systems hold promise in aiding prognostication for pleural infection, several challenges must be addressed to maximize their clinical utility. Firstly, these scoring systems' validation and external applicability pose significant concerns. Many of these systems are developed and validated in specific patient populations, which may limit their generalizability to diverse cohorts or healthcare settings. To ensure their reliability across various contexts, it is crucial to conduct rigorous validation studies in different patient populations representative of real-world scenarios [28].

Secondly, successfully integrating rapid scoring systems into routine clinical practice requires overcoming practical hurdles. Healthcare providers must receive adequate training and awareness regarding the use and interpretation of scoring results. Seamless incorporation into existing clinical workflows and overcoming potential resistance to change are essential steps in maximizing the impact of these scoring systems on patient care [29].

Another critical challenge is centered around predicting long-term outcomes. While rapid scoring systems often concentrate on short-term outcomes like hospital mortality or length of stay, complexity arises when predicting long-term outcomes, including factors like recurrence rates and functional recovery. These aspects demand a more extensive follow-up period and meticulous data collection. The development of scoring models that can reliably forecast these long-term outcomes holds significant importance, as it can greatly enhance patient management strategies and the support provided post-infection [30].

Addressing these challenges requires continued research and collaboration among healthcare institutions and researchers. External validation across diverse patient populations, transparent reporting of findings, and prospective studies can enhance the reliability and credibility of rapid scoring systems. Additionally, advances in data science and integrating novel biomarkers may offer opportunities to refine these scoring approaches, leading to more accurate and personalized prognostication for pleural infection [31].

By overcoming these challenges and refining existing rapid scoring systems, clinicians can make more informed decisions, tailor treatments, and allocate resources effectively, ultimately leading to improved patient outcomes and enhanced management of pleural infection. With ongoing efforts and future advancements, rapid scoring systems have the potential to revolutionize the way pleural infection is managed, providing valuable tools for healthcare providers and better care for patients [32].

Prognostic value of rapid scoring in pleural infection

Evaluating the Predictive Accuracy of Rapid Scoring Systems

Sensitivity and specificity analysis: Sensitivity measures the proportion of true positive cases correctly identified by the scoring system, i.e., the ability to classify patients with adverse outcomes correctly. Specificity, on the other hand, measures the proportion of true negative cases correctly identified, i.e., the ability to classify patients with favorable outcomes correctly. A comprehensive analysis of sensitivity and specificity provides valuable insights into the scoring system's ability to discriminate between patients with different outcomes. High sensitivity and specificity indicate a scoring system's ability to accurately identify positive and negative cases, thereby increasing its reliability in distinguishing patients with better or worse prognoses [33].

Receiver operating characteristic (ROC) curves: ROC curves graphically illustrate the trade-off between sensitivity and specificity at various score thresholds. By plotting sensitivity against (1 - specificity) for different threshold values, the ROC curve visually represents the scoring system's performance in distinguishing between patients with different outcomes. The area under the ROC curve (AUC) quantifies the overall discriminative ability of the scoring system. An AUC value close to 1 indicates excellent discriminative power, meaning the scoring system can effectively differentiate between patients with favorable and adverse outcomes. Conversely, an AUC value close to 0.5 suggests poor discrimination, equivalent to random chance, rendering the scoring system less effective in predicting patient outcomes [34]. Researchers and clinicians can rigorously evaluate the predictive accuracy of rapid scoring systems in pleural infection management by employing these key approaches. Systems with high sensitivity, specificity, and AUC values are more likely to be reliable and suitable for supporting clinical decision-making and risk stratification. These evaluations provide essential evidence for integrating rapid scoring systems into clinical practice, potentially improving patient care and outcomes in pleural infection management.

Predictive Potential for Short-Term Outcomes

Mortality prediction: Mortality prediction is a critical aspect of managing pleural infection, as identifying patients at higher risk of death enables healthcare providers to initiate timely interventions and allocate resources effectively. By integrating clinical and laboratory parameters, rapid scoring systems aim to quantify the mortality risk in infected patients. The scores generated by these systems can serve as an early warning system, alerting clinicians to patients with a higher likelihood of adverse outcomes. Studies that analyze the association between rapid scoring system scores and actual mortality rates are of paramount importance. Evaluating the accuracy and reliability of these scoring systems in predicting mortality is essential for their successful implementation in clinical practice. Validated scoring systems can support evidence-based clinical decision-making, guiding aggressive treatment approaches for high-risk patients and potentially improving overall survival rates in pleural infection cases [15].

Treatment response: Assessing the predictive potential of rapid scoring systems on treatment response is equally significant. Identifying patients less likely to respond to standard therapies is crucial for early intervention and alternative treatment strategies. Rapid scoring systems can assist in predicting treatment response by considering factors such as disease severity, pathogen characteristics, and patient-specific variables. By flagging non-responsive or slow-responding cases, clinicians can promptly modify treatment regimens, thereby preventing disease progression and minimizing complications. This proactive approach to treatment tailoring may improve therapeutic efficacy and enhance patient outcomes. Furthermore, predicting treatment response can aid in the judicious use of healthcare resources, optimize medication utilization, and reduce unnecessary interventions [35].

Length of hospital stay: Prolonged hospital stays in pleural infection cases impact patient well-being and impose a substantial economic burden on healthcare systems. Rapid scoring systems that accurately predict the length of hospitalization can provide valuable insights for resource management and discharge planning. By estimating the expected duration of hospital stay, healthcare providers can allocate resources more efficiently, including hospital beds, staff, and medications. This allows for smoother patient flow and reduces overcrowding in medical facilities. Additionally, accurate predictions of hospital stay duration enable healthcare teams to engage in proactive discharge planning, ensuring that patients receive appropriate care and support after discharge. By minimizing unnecessary hospitalization, rapid scoring systems can contribute to optimizing healthcare resources and improve overall patient care in the context of pleural infection management [36,37].

Predictive Potential for Long-Term Outcomes

Recurrence rates: Pleural infection recurrence is a vexing issue that poses challenges in patient management. Identifying individuals at higher risk of recurrence is crucial for implementing appropriate surveillance and follow-up strategies. Rapid scoring systems, with their ability to provide a quantitative assessment of disease severity, may offer valuable insights into the likelihood of recurrence. Clinicians can tailor long-term treatment planning and preventive measures for high-risk patients by analyzing the correlation between scoring system scores and recurrence rates. A scoring system capable of predicting recurrence can prompt intensified monitoring, early interventions, or targeted therapies to reduce the burden of recurrent pleural infections and improve patient outcomes [38,39].

Functional outcomes and quality of Life: Beyond short-term outcomes such as mortality and recurrence rates, the impact of pleural infection on functional outcomes and quality of life is paramount. Many survivors of pleural infection experience impaired lung function, reduced exercise capacity, and diminished overall quality of life. Scoring systems that can predict long-term functional outcomes may prove instrumental in optimizing rehabilitation efforts and post-infection support. By identifying patients at risk of poor functional recovery, clinicians can initiate timely interventions such as pulmonary rehabilitation, physical therapy, and psychological support to enhance the patient's overall well-being and quality of life [40,41].

Understanding the predictive value of rapid scoring systems for both short-term and long-term outcomes is essential for their effective clinical application. The analysis of various studies investigating these aspects provides a comprehensive overview of the potential benefits and limitations of using rapid scoring systems in pleural infection management. Integrating rapid scoring into clinical practice can facilitate risk stratification, personalized treatment planning, and informed decision-making. However, it is crucial to recognize these scoring systems' limitations and potential biases and address them through rigorous validation and future research. By refining and enhancing the accuracy and utility of rapid scoring systems, healthcare providers can optimize patient care and improve outcomes in pleural infection management. Ultimately, the successful integration of rapid scoring into routine clinical practice can positively impact patient well-being and healthcare resource allocation, bringing us closer to more effective and patient-centered pleural infection management strategies [15,21].

Factors affecting the prognostic accuracy of rapid scoring

Accurate prognostication is a critical aspect of managing pleural infection effectively. Rapid scoring systems offer a quantifiable and standardized approach to assessing disease severity and predicting patient outcomes. However, to ensure their reliability and usefulness in clinical practice, it is essential to consider various patient-specific factors that can influence the predictive accuracy of these scoring systems [42]. One such factor is patient demographics. Age and gender, for instance, can significantly impact disease progression and treatment response in pleural infection. Elderly patients or those with certain comorbidities may have a higher risk of adverse outcomes. By accounting for these demographic factors in the scoring models, clinicians can better tailor their treatment plans and allocate resources accordingly [43].

Microbiological and radiological findings are another crucial aspect of pleural infection management. The causative microorganisms, their antibiotic sensitivity, and the extent of lung involvement and pleural effusion on imaging can significantly affect disease severity and treatment response. Rapid scoring systems incorporating these findings into their algorithms may offer more accurate prognostic predictions [44]. Comorbidities, such as diabetes, heart disease, or immunosuppression, can complicate the clinical course of pleural infection and influence patient outcomes. Scoring systems considering comorbid conditions can provide a more comprehensive assessment of the patient's overall health status, enabling clinicians to make more informed decisions regarding treatment and follow-up care [45].

Moreover, interpreting scoring results in the context of the severity of pleural infection is crucial. In mild or moderate cases, scoring systems may have limited discriminatory ability due to the relatively uniform nature of the patient population. However, scoring systems can offer more accurate prognostic insights in severe or complicated infections, guiding clinicians towards more aggressive interventions and monitoring strategies [46].

Impact of Patient Demographics

Patient demographics, specifically age and sex, influence disease progression and outcomes in pleural infection. Age-related factors can significantly impact the clinical course of the infection. As patients age, changes in immune function and physiological reserves may increase infection vulnerability, making elderly individuals more susceptible to severe pleural infection. Additionally, comorbidities commonly accompanying aging can complicate disease management and influence treatment response. Therefore, considering age-related variations in prognostic scoring systems is essential to accurately predict disease severity and outcomes in different age groups [47].

Furthermore, gender-related differences in pleural infection outcomes have been observed in some studies. While the underlying reasons for these disparities are not yet fully understood, it is believed that hormonal and immunological differences between males and females may contribute to varying disease outcomes. For instance, estrogen is known to have immunomodulatory effects that might influence the body's response to infection. Some studies have suggested that female patients with pleural infection may experience milder disease courses and have better treatment responses than males. By incorporating gender as a parameter in prognostic scoring systems, it is possible to account for these gender-specific differences and improve the accuracy of predicting outcomes for both male and female patients [48].

Addressing patient demographics in rapid scoring systems is essential for personalized medicine and individualized patient care. Tailoring treatment strategies based on age and gender considerations can help clinicians optimize therapeutic interventions and improve patient outcomes. However, it is crucial to ensure that any consideration of demographics in scoring systems is evidence-based and supported by rigorous research. Understanding the impact of age and gender on pleural infection outcomes is an ongoing area of investigation, and continued research in this domain will further enhance the accuracy and utility of rapid scoring systems in pleural infection prognostication [49].

Role of Microbiological and Radiological Findings

Microbiological and radiological findings are integral components in diagnosing and managing pleural infection. Identifying causative microorganisms and their susceptibility to antibiotics is crucial for guiding appropriate antimicrobial therapy, significantly impacting treatment response and overall patient outcomes. Rapid scoring systems that consider microbiological data have the potential to enhance predictive accuracy, particularly in cases where tailored antibiotic treatment is necessary [50].

Microbiological data can reveal valuable information about the type of pathogen causing the infection, its virulence, and its antibiotic resistance. Scoring systems that incorporate microbiological findings can aid clinicians in identifying high-risk patients who may require prompt escalation to targeted and more aggressive therapies. This enables timely adjustments to treatment regimens, potentially preventing treatment failure and the development of complications [51].

Conversely, evaluating disease severity and tissue involvement heavily relies on radiological characteristics. Essential insights regarding pleural effusion, lung parenchymal engagement, and necrosis are provided through imaging techniques like chest X-rays and computed tomography (CT) scans. These radiological attributes mirror both the local and systemic effects of the disease, thereby enhancing clinicians' comprehension of the disease's status and possible complications [52].

Scoring systems integrating radiological parameters offer a more comprehensive and nuanced disease evaluation. Such assessments can assist in risk stratification, allowing healthcare providers to identify patients with more severe infections who may require more intensive management, such as drainage procedures or surgical interventions [53]. By considering both microbiological and radiological findings, rapid scoring systems can provide a holistic approach to prognostication and guide tailored therapeutic strategies for individual patients. Including these critical diagnostic components strengthens the predictive value of the scoring systems, thereby enhancing their clinical utility and supporting more informed decision-making in pleural infection management.

Influence of Comorbidities on Scoring Systems

Comorbidities significantly shape the clinical course of pleural infection, influencing disease progression, treatment response, and patient outcomes. Conditions such as diabetes, chronic kidney disease, heart failure, and immunosuppressive states can weaken the immune system, impair lung function, and increase the risk of complications. Consequently, patients with comorbidities are often at higher risk of adverse outcomes, including prolonged hospital stays, treatment failure, and increased mortality [54].

Incorporating comorbidities into rapid scoring systems can enhance their prognostic accuracy by providing a more comprehensive assessment of the patient's overall health status. Scoring models considering these additional risk factors can better identify high-risk patients requiring more aggressive interventions or closer monitoring. Tailored treatment plans based on accurate risk stratification can improve clinical outcomes and resource utilization, ensuring that the most vulnerable patients receive the necessary care [55]. However, a balance must be struck in including comorbidities in scoring systems. Incorporating too many variables can lead to excessive complexity, making the scoring calculations cumbersome and time-consuming. In busy clinical settings, rapidity is essential for decision-making, and scoring systems should be designed for ease of use and rapid calculation.

Additionally, choosing which comorbidities to include in the scoring systems must be carefully considered. Not all comorbidities may impact pleural infection outcomes equally, and some may be more relevant in specific patient populations. Thus, the selection of comorbidities should be based on their clinical significance and the availability of data [56]. Moreover, as new evidence emerges, the inclusion of comorbidities in scoring systems should be periodically reevaluated and updated. Advancements in medical knowledge may highlight previously unrecognized risk factors or indicate that certain comorbidities are less relevant than initially assumed. Regular review and refinement of scoring systems will ensure their relevance and accuracy in contemporary clinical practice [57].

Interpreting Scoring Results in the Context of Pleural Infection Severity

The interpretation of scoring results in the context of pleural infection is critical when utilizing rapid scoring systems. It is important to recognize that these scoring systems' accuracy and predictive power may vary depending on the overall severity of the pleural infection in a given patient population [14]. The disease manifestations and outcomes may be relatively homogenous in patients with mild or moderate pleural infection. As a result, the scoring system may have limited discriminatory ability in distinguishing between different risk categories or predicting specific outcomes. In such cases, clinicians should exercise caution and consider other clinical factors besides the scoring results to make well-informed decisions [40]. On the other hand, in severe or complicated cases of pleural infection, the disease may manifest with a wider spectrum of clinical presentations and outcomes. In these scenarios, the rapid scoring system may demonstrate improved accuracy in predicting adverse outcomes, such as mortality or treatment failure. In such situations, rapid scoring can be particularly valuable in identifying patients who require immediate and aggressive interventions [3].

Despite their potential usefulness, clinicians need to understand that rapid scoring systems are adjunctive tools and not definitive predictors of outcomes. These systems are designed to aid in risk stratification and decision-making but should not be solely relied upon to make critical clinical decisions. Instead, they should be used with clinical judgment and other diagnostic assessments, such as radiological findings, microbiological data, and the patient's overall clinical status [15]. By integrating rapid scoring results with other clinical information, clinicians can create a more comprehensive picture of the patient's condition and tailor their management plan accordingly. This collaborative approach ensures a more holistic and patient-centered decision-making process, optimizing the care and outcomes for individuals affected by pleural infection. Ultimately, rapid scoring systems serve as valuable tools to guide clinical decisions, but the expertise and experience of the healthcare team remain essential in delivering optimal patient care [58].

Clinical implications and application of rapid scoring

Role of Rapid Scoring in Clinical Decision-Making

Rapid scoring systems in pleural infection management have the potential to significantly impact clinical decision-making by providing a quantitative and standardized assessment of disease severity and prognostication. These scoring systems offer valuable information to healthcare providers, aiding in risk stratification, treatment tailoring, and follow-up planning [59].

Risk stratification: Rapid scoring systems excel in identifying patients at higher risk of adverse outcomes, such as mortality or treatment failure. These systems can efficiently classify patients into risk categories by analyzing clinical and laboratory parameters. This enables clinicians to prioritize interventions and allocate resources effectively, especially in resource-constrained settings where optimal utilization of healthcare resources is paramount [55].

Treatment tailoring: One of the key challenges in pleural infection management is selecting the most appropriate treatment strategies for individual patients. Rapid scoring systems play a pivotal role by providing insights into disease severity and potential response to treatment. The scoring results can guide clinicians in choosing the most suitable antimicrobial therapy and drainage procedures and tailoring treatment plans based on the predicted disease severity. This personalized approach enhances therapeutic efficacy, minimizes the risk of treatment failure, and reduces the likelihood of complications [5].

Follow-up planning: Beyond short-term prognostication, rapid scoring systems offer valuable information for long-term follow-up planning. By predicting disease trajectory and long-term outcomes, such as recurrence rates, these systems can inform healthcare providers about the need for post-discharge care and surveillance. Timely detection of potential complications and recurrent infections allows for proactive intervention, leading to better disease management and patient outcomes [60].

Integrating rapid scoring systems into clinical practice empowers healthcare providers with evidence-based decision-making tools, aiding in more informed and precise patient care. The ability to quickly assess disease severity, predict outcomes, and tailor treatments based on individual patient characteristics enhances the overall management of pleural infection, ultimately leading to improved patient outcomes and enhanced healthcare resource utilization. However, ongoing research and validation are essential to continually refine and optimize these scoring systems, ensuring their effectiveness and reliability in real-world clinical settings. By harnessing the full potential of rapid scoring systems, healthcare providers can revolutionize pleural infection management and elevate the standard of care for patients affected by this challenging condition [61].

Integration of Rapid Scoring into Current Management Guidelines

Validation and adaptation: Thorough validation is essential before implementing rapid scoring systems. This involves evaluating the performance of the scoring system in diverse patient populations to ensure its generalizability across different contexts. Validation studies should encompass various demographic profiles, disease severities, and geographic locations. In some cases, adaptation of the scoring system to specific local contexts may be necessary to account for variations in patient characteristics and healthcare practices [62].

Education and training: Effective education and training programs are crucial to familiarize healthcare providers with the rapid scoring system's use and interpretation. Clinicians need to understand the rationale behind the scoring criteria, the significance of specific variables, and how to calculate the scores accurately. Additionally, emphasizing that the scoring system is an adjunctive tool to support clinical judgment can help mitigate any concerns of over-reliance on the scoring results [63].

Standardized documentation: Consistent and standardized documentation of scoring system parameters in patient records is vital for continuity of care. Healthcare providers should diligently record the data used to calculate the score, the actual score itself, and any updates during treatment. Standardized documentation enables clinicians to track disease progression over time and aids in assessing the effectiveness of treatment strategies based on changes in the scoring results [64].

Interdisciplinary collaboration: Pleural infection management involves the collaboration of various medical specialists, each contributing their expertise to patient care. Effective interdisciplinary collaboration is essential for integrating rapid scoring systems seamlessly into the overall management approach. Collaborating with pulmonologists, infectious disease specialists, radiologists, and other relevant disciplines like cardiothoracic ensures that all aspects of patient care are considered, and the scoring system's results are interpreted comprehensively [65].

Practical Considerations and Challenges in Implementing Rapid Scoring

Accessibility of data: The availability of necessary clinical and laboratory data required for rapid scoring may vary across different healthcare settings. In some facilities, electronic health records (EHRs) might be well-established, allowing easy access to relevant patient data. However, in resource-limited or smaller healthcare facilities, the absence of comprehensive EHR systems could hinder data accessibility. Efforts should be made to streamline data collection and ensure the completeness of data inputs for accurate scoring. This may involve improving data capture and recording practices, and fostering interoperability between healthcare information systems [66].

Scoring system selection: Choosing the most appropriate rapid scoring system for a specific clinical setting requires careful consideration of various factors. Different scoring systems may have been developed and validated in diverse patient populations or healthcare contexts. As such, it is essential to evaluate the applicability and accuracy of each scoring system in the specific patient cohort and resource constraints of the healthcare facility. Factors such as patient demographics, available resources, and the overall clinical context should be considered during the selection process to ensure that the chosen scoring system aligns with the needs and capabilities of the healthcare setting [67].

Time constraints: Rapid scoring systems are designed to provide quick and efficient disease severity and prognosis assessments. However, the practicality of their implementation can be impacted by time constraints, particularly in busy clinical settings where healthcare professionals have limited time to dedicate to scoring calculations. The scoring process should be user-friendly, intuitive, and time-efficient to integrate rapid scoring into routine clinical practice seamlessly. Implementing user-friendly electronic scoring tools or incorporating scoring algorithms into electronic medical record systems can help minimize the time investment required for scoring calculation [21].

Future Directions and Potential Improvements in Rapid Scoring Systems

Comprehensive scoring models: To enhance the accuracy of prognostication, future scoring systems could be developed as more comprehensive models. By incorporating additional variables, such as molecular biomarkers or novel radiological parameters, these systems can provide a more nuanced and detailed assessment of disease severity and progression. Integrating biomarkers reflecting the host's immune response or pathogen virulence may offer valuable insights into disease outcomes and treatment responses [68].

Artificial intelligence (AI) integration: Leveraging AI algorithms could revolutionize rapid scoring systems by analyzing vast datasets and identifying patterns that might not be apparent through conventional methods. AI integration can significantly improve the predictive power of these systems. Machine learning algorithms can adapt and refine scoring models based on real-world data, ensuring continuous improvement and adaptability to evolving clinical scenarios [69].

External validation and standardization: It is essential to rigorously validate and standardize rapid scoring systems across diverse patient populations and healthcare settings for widespread clinical adoption. Continued research and collaboration among healthcare institutions and research centers can facilitate the validation process, ensuring the reliability and generalizability of these scoring approaches [70].

Longitudinal predictions: Advancements in scoring methodologies may enable dynamic and longitudinal predictions, offering real-time updates on patient outcomes throughout treatment. Longitudinal assessments can provide insights into disease progression, response to therapies, and potential complications, allowing clinicians to adjust treatment strategies proactively [71].

Top of form critique and limitations of rapid scoring systems

Criticism of Current Scoring Approaches

While rapid scoring systems have demonstrated their potential in aiding prognostication for pleural infection, they are not exempt from criticism. Several key criticisms of current scoring approaches warrant consideration:

Oversimplification: One significant critique of some rapid scoring systems is their tendency to oversimplify the complexity of pleural infection. By focusing on a limited set of parameters, these scoring systems may not fully capture the multifaceted nature of the disease. The oversimplification could lead to inadequate prognostic accuracy, particularly in cases with diverse clinical presentations and variable disease trajectories [14].

Generalizability: Many rapid scoring systems have been developed and validated in specific patient populations, often based on data from single institutions or regions. Consequently, there may be concerns regarding the applicability of these scoring approaches to more diverse patient cohorts. The lack of external validation across different healthcare settings and populations hinders their generalizability and raises questions about their reliability in real-world clinical scenarios [72].

Lack of longitudinal assessment: Rapid scoring systems typically provide static assessments of disease severity at a particular time point. The absence of dynamic or longitudinal assessments limits their ability to predict changes in disease trajectory over time. In pleural infection, disease progression and response to treatment may evolve over days or weeks, and a single snapshot may not capture the full clinical picture [73].

Limited predictive value for long-term outcomes: While rapid scoring systems have shown promise in predicting short-term outcomes, such as mortality and treatment response, their ability to predict long-term outcomes remains a challenge reliably. Variables influencing long-term recovery, recurrence rates, and functional outcomes are complex and multifactorial, making their inclusion in scoring systems more complex [74].

Limitations of Available Studies and Data

Small sample sizes: Many studies evaluating rapid scoring systems may have limited sample sizes due to the rarity of severe pleural infections or other logistical constraints. Small sample sizes can reduce statistical power, making it challenging to draw definitive conclusions. Moreover, findings from such studies may lack generalizability to larger, more diverse patient populations, potentially limiting the external validity of the scoring systems [75].

Heterogeneity of studies: Studies evaluating rapid scoring systems often utilize varying patient populations, methodologies, and outcome measures. This heterogeneity can complicate direct comparisons between different scoring approaches, hindering the establishment of a consensus on the most effective scoring system for pleural infection prognostication. A standardized approach to study design and outcome measurements would facilitate meaningful comparisons and enhance the reliability of the findings [76].

Retrospective nature: Many studies assessing rapid scoring systems in pleural infection rely on retrospective data, which can introduce inherent biases and limitations. Retrospective data collection may be subject to selection bias, as patient data may not have been originally collected for scoring system evaluation. Additionally, retrospective data might lack completeness, leading to missing or incomplete information that could impact the accuracy and reliability of prognostic evaluations [77].

Missing data and follow-up: Incomplete data and loss to follow-up present significant challenges in prognostic evaluations using rapid scoring systems. Missing data can result from various factors, including patients' refusal to participate in follow-up assessments or incomplete medical records. This missing data can lead to underestimation or overestimation of prognostic accuracy and potentially introduce bias into the results. Ensuring complete and accurate data collection, as well as adequate follow-up, is essential to minimize these potential biases and enhance the validity of prognostic assessments [78].

Addressing Potential Sources of Bias

Large, prospective multicenter studies: Conducting well-designed prospective studies with large and diverse patient populations is essential. By collecting data from multiple healthcare centers, these studies can improve the generalizability of the findings and ensure that the results represent the broader patient population. Prospective studies also allow for standardized data collection, minimizing the risk of data inconsistencies and enhancing the statistical robustness of the scoring system [72].

External validation: External validation of rapid scoring systems is critical to establishing their predictive accuracy in different clinical settings and patient cohorts. Validating the scoring systems in settings beyond their original development ensures that they perform consistently and reliably across diverse healthcare contexts. Such validation strengthens the evidence base supporting the use of these scoring systems and increases confidence in their clinical applicability [79].

Longitudinal assessment: Incorporating longitudinal assessments into rapid scoring systems can provide more accurate predictions of disease trajectories over time. By tracking changes in patient status and outcomes throughout treatment and follow-up, longitudinal assessments can offer insights into the dynamic nature of pleural infection and its response to interventions. This temporal dimension can further enhance the predictive value of the scoring systems, guiding clinicians in making more informed decisions throughout the patient's healthcare journey [80].

Transparent reporting: Transparent reporting of study methods, data collection, and potential sources of bias is crucial for ensuring the credibility and reproducibility of research findings. Comprehensive and clear reporting allows other researchers and healthcare professionals to understand and evaluate the study's methodology and potential limitations. It fosters an environment of scientific rigor and encourages transparency in research, which is vital for building trust in the validity and applicability of rapid scoring systems in pleural infection prognostication [81].

## Conclusions

This review article has comprehensively assessed the predictive potential of rapid scoring systems in pleural infection prognostication. Pleural infection is a significant health concern, demanding accurate prognostic tools for effective management. Our analysis of existing scoring systems such as CURB-65, A-DROP, and APACHE II revealed their strengths and limitations in predicting short-term and long-term outcomes. While rapid scoring systems show promise in aiding clinical decision-making and risk stratification, they are not without drawbacks and face challenges related to data limitations and potential biases. However, their integration into clinical practice can potentially improve patient outcomes and optimize resource allocation. Moving forward, rigorous validation, reporting transparency, and data science advances offer promising avenues for refining rapid scoring systems. Multidisciplinary collaboration and prospective studies are critical to unlocking the full potential of rapid scoring in pleural infection management, ultimately contributing to better patient care and outcomes.
